# Participatory sport consumption as capability building in later life: a framework for competence, connectedness, and active ageing

**DOI:** 10.3389/fpsyg.2026.1913428

**Published:** 2026-10-23

**Authors:** Zhe Zhang, Zhi jie Lai, Xiaohong Wan

**Affiliations:** 1 School of Journalism and Communication, Wuhan Sports University, Wuhan, China; 2 Sports Social Science Research Center of the General Administration of Sport of China, Wuhan Sports University, Wuhan, China; 3 School of Physical Education, Guangzhou College of Commerce, Guangzhou, China

**Keywords:** active ageing, capability approach, capability building, competence, connectedness, older adults, participatory sport consumption

## Abstract

This paper develops the concept of participatory sport consumption in later life, referring to older adults’ acquisition, access, use, and combination of sport-related resources that support their own participation in sport and exercise. Drawing on the Capability Approach, the paper distinguishes resource availability from the substantive opportunities older adults are able to exercise and value. The proposed framework identifies personal, socioeconomic and relational, and environmental and service conditions as determinants of resource convertibility. Mastery, agency, and relational-identity conversion are specified as capability-building processes, with competence and connectedness as proximal manifestations and later-life functionings as possible outcomes. The framework also incorporates capability failure, recognising that access or participation may coexist with restricted opportunity. It provides a basis for examining how sport-related resources contribute to capability expansion and inequality in later-life participation.

## 1 Introduction

Population ageing has intensified interest in forms of later-life participation that support health, autonomy, social relationships, and meaningful engagement. Sport occupies a distinctive position within this agenda because bodily activity is embedded in experiences of achievement, enjoyment, interaction, and continuing involvement in collective life. A systematic review of 21 studies involving adults aged 50 years and older identified outcomes including life satisfaction, socialisation, empowerment, and self-confidence (Kim et al., 2020). A review of 30 studies involving adults aged 55 years and older further showed that health, supportive social environments, team affiliation, achievement, competition, and travel commonly motivated continued sport participation (Stenner et al., 2020). Survey evidence from 600 Spanish adults aged 50–70 also associated active sport engagement with stronger social networks and greater satisfaction with personal relationships (Sánchez-Santos et al., 2024). Sport in later life therefore encompasses bodily, psychological, and relational experiences that cannot be captured by physical-activity volume alone.

Research on later-life sport has primarily explained participation and its determinants. A systematic review of community-dwelling older adults identified health status, previous sport experience, social connection, and opportunities for meaningful involvement as important influences on continued participation (Jenkin et al., 2017). A socioecological study of adults aged 50 years and older located participation within interacting individual, interpersonal, organisational, community, and policy conditions (Chen et al., 2023). Socioeconomic differences further shape these opportunities: recent evidence indicates that the correlates and determinants of physical activity vary according to socioeconomic position, placing access to resources and structural inequality within the explanation of participation itself (Malkowski et al., 2025). This literature explains substantial variation in whether and under what conditions older adults participate, while giving less analytical attention to the goods, services, technologies, facilities, and organisational arrangements through which participation is made possible.

Such resource arrangements are integral to many forms of later-life sport. Participation may depend on facility access, memberships, coaching, equipment, transport, rehabilitation-oriented services, digital technologies, and organised programmes. Qualitative research involving Australian sport organisations, sport-club members, and non-club adults aged 50 years and older found that the availability and suitability of organised opportunities shaped continued involvement alongside health and social considerations (Jenkin et al., 2018). In the United States, comparison of older adults enrolled in the SilverSneakers programme with matched non-members linked membership-based access with differences in physical activity and social-health indicators (Brady et al., 2020). Sport-consumer research also distinguishes participant and spectator markets because personal participation involves behavioural processes and motivations that differ from those associated with watching sport (McDonald et al., 2002). These findings locate sport-related resources and service arrangements as a substantive component of participation rather than as peripheral consumption surrounding an otherwise independent behaviour.

Physical activity, sport participation, and the resource arrangements supporting participation consequently represent different analytical objects. Physical activity refers conventionally to bodily movement that produces energy expenditure, with exercise constituting a planned and structured subset (Caspersen et al., 1985). Sport participation embeds such activity within sporting practices that can generate social and psychological experiences alongside physical outcomes; systematic evidence has associated sport participation with social interaction, belonging, wellbeing, and psychological benefits (Eime et al., 2013). Research among adults aged over 65 similarly identifies cognitive, emotional, social, and motivational outcomes while cautioning against attributing them automatically to sport participation itself (Gayman et al., 2017). Participatory sport consumption in later life (PSCILF) is located at the resource level of this relationship. It refers to older adults’ acquisition, access, use, and combination of sport-related goods, services, technologies, facilities, and organised settings that directly enable, structure, support, or sustain their own participation in sport and exercise. PSCILF is therefore distinct from the physical activity performed, the participation behaviour observed, and spectator-oriented forms of sport consumption. Its later-life specificity concerns how sport-related resources interact with changing bodily, socioeconomic, and relational circumstances across older adulthood. Although similar products and services may be used across age groups, differences in functional capacity, retirement circumstances, financial resources, and social relationships may affect their accessibility, usability, and personal value. These conditions vary across individuals and shape the opportunities that sport consumption can provide in later life.

Resource access does not determine what older adults can achieve through participation. A meta-analysis of 25 quantitative studies involving middle-aged and older adults found substantial heterogeneity in the psychosocial outcomes associated with sport participation, indicating that exposure to sport does not generate uniform benefits (Sivaramakrishnan et al., 2024). Opportunities are also unequally distributed. A 15-year population study in Geneva documented persistent socioeconomic inequalities in sport participation and increasing income-related differences among some groups, including older adults (Richard et al., 2023). Technology introduces another layer of variation: a systematic review and meta-analysis of digitally delivered home-based exercise for older adults reported potential benefits alongside considerable heterogeneity in intervention characteristics and outcomes (Solis-Navarro et al., 2022). Formal availability of a programme, membership, facility, or technology therefore provides information about resources, but not about the substantive opportunities those resources create for a particular older adult. Service design and institutional arrangements may further influence whether these resources accommodate diverse bodily circumstances and enable meaningful participation choices.

The Capability Approach offers an analytical basis for explaining this gap between resources and substantive opportunity. Its relevance to sport is supported by applications that place agency, institutional organisation, social relations, and environmental conditions within the evaluation of participation. A capability-based analysis of sport for social inclusion conceptualised sporting programmes as settings whose contribution depends on interactions among individual agency, organisational processes, and wider social structures (Suzuki, 2017). Qualitative research with adults aged 66–97 in Southern Germany identified physical-activity capabilities across individual resources, social relations and norms, living conditions, and organisational environments, demonstrating that opportunities for active living depend on more than personal willingness (Sauter et al., 2019). Interviews with adults experiencing poverty and social exclusion in a German voluntary sports club further showed that flexible participation, reliable programme structures, and supportive group cultures operated as conversion conditions associated with coping, belonging, and the capacity to influence physical and mental change (Oettle and Greiner, 2025). The Capability Approach provides a basis for examining why similar sport-related resources generate different substantive opportunities across older consumers. By treating service organisation and institutional arrangements as conversion conditions, it also enables the analysis of participation restrictions that arise from the interaction between individual circumstances and the organisation of sport provision.

This paper develops a capability-centred framework to explain how PSCILF resources become feasible and valued participation opportunities for older adults. It examines the conversion conditions and capability-building processes through which sport-related resources may support competence, connectedness, and valued later-life functionings, while identifying the individual and institutional conditions under which capability expansion may be restricted.

## 2 Conceptualising participatory sport consumption in later life

### 2.1 Conceptual location of PSCILF

Sport consumption extends beyond the purchase of sporting goods. Sport-consumer research conceptualises consumption as engagement with the products and services through which sport is accessed and experienced, encompassing multiple stages and forms of consumer behaviour rather than purchase alone (Funk et al., 2003). Research on participant sport services similarly identifies programmes, staff interaction, physical environments, equipment, and perceived outcomes as interconnected components of the consumption experience (Ko and Pastore, 2004). Sport consumption encompasses the acquisition and use of sport-related resources and services, including the arrangements through which participation is accessed and experienced.

Participant-oriented and spectator-oriented consumption occupy different positions within this broader domain. Research in Japan comparing spectators at a professional baseball game with users of a running station found that both constituted sport-consumption settings, while the relevant service attributes and consumer responses differed between spectator and participant contexts (Masayuki and Makoto, 2016). Spectator consumption can itself generate positive emotions, social relationships, meaning, and engagement, as shown in research on Australian Rules Football spectators (Doyle et al., 2016). These benefits, however, do not depend on the consumer’s own physical participation. PSCILF concerns sport-related resources that directly support older adults’ own participation in sport and exercise. Spectator-oriented consumption falls outside this construct because its primary activities do not require the consumer’s own physical participation.

Sport-related resources constitute the means through which participation may be enabled or organised, whereas physical activity and sport participation describe forms of action that may subsequently occur. PSCILF shares the resource and service characteristics of general participatory sport consumption, while its analytical specificity lies in how these resources interact with the changing and heterogeneous circumstances of later life.

### 2.2 Defining participatory sport consumption in later life as resource-mediated participation

PSCILF refers to older adults’ acquisition, access, use, and combination of sport-related goods, services, technologies, facilities, and organised settings that directly enable, structure, support, or sustain their own participation in sport and exercise. Four analytical conditions delimit the construct.

First, an identifiable sport-related resource or service arrangement must be involved. Second, that resource must bear a direct functional relationship to the individual’s own physical participation. Third, consumption includes access and use as well as private purchase, allowing publicly funded, community-based, subsidised, and commercial provision to fall within the construct. Fourth, resource possession or use remains analytically distinct from the opportunities, experiences, and outcomes that may arise through participation. Everyday physical activity alone does not constitute PSCILF unless an identifiable sport-related resource or service arrangement directly enables, organises, or supports the individual’s sport or exercise practice.

PSCILF encompasses older adults who acquire or use sport-related resources to organise their own participation across commercial, public, community-based, and subsidised settings. Later life is treated as a life-course context rather than a uniform chronological or functional category, recognising that older adults differ considerably in health, socioeconomic resources, previous sport experience, and participation preferences. Compared with general sport-consumption analysis, PSCILF places particular analytical attention on how functional changes, retirement circumstances, financial resources, and evolving social relationships may affect the accessibility, usability, and personal value of sport-related resources. These conditions vary across individuals and influence whether similar consumption arrangements generate feasible and valued participation opportunities. The Capability Approach provides an analytical basis for examining these differences by distinguishing the resources available to older consumers from the substantive opportunities they can exercise. Its emphasis on conversion factors and agency enables the evaluation of whether sport-related provision accommodates heterogeneous later-life circumstances and preserves meaningful participation choices.

Evidence from participant sport services supports this separation between resource arrangements and subsequent outcomes. Research involving 2,109 users of eight public aquatic centres in Australia distinguished programme processes, perceived outcomes, value, and satisfaction within the participant-service experience (Howat and Assaker, 2016). A study of 3,186 marathon participants likewise found that service quality, perceived economic value, and athletic goal achievement made different contributions to satisfaction, intention to participate again, and future exercise intention (Hyun and Jordan, 2020). These findings support the analytical separation of sport-related resources, participation experiences, and subsequent outcomes within PSCILF.

Evidence from 4,837 Swedish adults aged 60–96 participating across more than 50 organised sports shows that later-life sport participation derives meaning from combinations of enjoyment, social relationships, routine, embodied satisfaction, self-worth, and perceived capability (Geidne et al., 2026). These findings illustrate the heterogeneous meanings older adults attach to organised participation and provide a basis for examining how sport-related resource arrangements can accommodate different participation goals and valued experiences.

### 2.3 Participation formats and resource configurations

Resource use can be organised through individual, family-based, or group-based participation. These formats represent different social configurations of participation rather than fixed categories of consumers. Individual formats may permit greater control over timing, pace, intensity, and self-monitoring. Group-based settings add relational resources such as peer observation, repeated interaction, shared routines, instructor contact, mutual recognition, and opportunities to occupy continuing social roles. The capability value of each format depends on whether its resource configuration accommodates the participant’s bodily circumstances, social preferences, and desired level of independence and interaction.

Evidence from older populations illustrates the relevance of this distinction. A study of 78,002 community-dwelling Japanese adults aged 65 years and older across 31 municipalities found that regular participation in sports groups was associated with socioeconomic, psychosocial, social, and environmental factors, including emotional support, social networks, and access to facilities (Yamakita et al., 2015). Qualitative research embedded in the GOAL trial in Canada showed that age-matched group exercise could generate supportive peer interaction while also producing challenges related to social comparison, isolation, and understandings of the ageing body (Bennett et al., 2018). Research involving adults aged 55 years and older across four recreation facilities further demonstrated that group physical activity could support social contact, belonging, relationship formation, and regular engagement when programme organisation facilitated sustained interaction (Zimmer et al., 2021). These findings indicate that group participation creates different relational opportunities according to programme organisation, peer interaction, and participants’ experiences of inclusion or social comparison.

PSCILF frequently involves resource configurations combining equipment, facilities, professional support, digital technologies, and social arrangements. The capability value of these configurations depends on whether their components can be accessed, coordinated, and adapted to older adults’ heterogeneous circumstances and participation goals. Resource configuration therefore provides an analytical basis for examining how individual and group-based participation may generate different substantive opportunities in later life.

## 3 The capability approach in later-life sport consumption

### 3.1 Core evaluative logic of the capability approach

The Capability Approach emerged from Amartya Sen’s work on welfare, development, and social justice. Its central evaluative concern is the substantive freedom people have to pursue ways of living they have reason to value. Resources and achieved behaviours are relevant, but neither provides a sufficient account of wellbeing because individuals differ in their ability to transform available means into valued forms of living (Sen, 1999).

This logic is organised around four related concepts: resources, conversion factors, capabilities, and functionings. Resources refer to the goods, services, technologies, infrastructure, and institutional arrangements available to individuals. Conversion factors describe the personal, social, and environmental conditions that affect whether those resources become usable opportunities. Capabilities represent the genuine opportunities available to a person, whereas functionings refer to the activities and states that are actually realised (Robeyns, 2005). Formally similar resources can therefore generate substantially different opportunities when individuals differ in bodily circumstances, socioeconomic position, social relations, or environmental conditions. Institutional arrangements and service practices also shape this conversion by influencing which opportunities are accessible, adaptable, and practically available to different individuals.

Nussbaum develops this perspective by identifying central capability domains including bodily health, bodily integrity, affiliation, play, and control over one’s environment. These domains are particularly relevant to later-life sport because they connect bodily activity with enjoyment, social recognition, and practical influence over everyday life (Nussbaum, 2011). They are used here as interpretive domains rather than as a fixed standard of desirable ageing.

The Capability Approach consequently prioritises substantive opportunity rather than resource possession or realised functioning alone. Its application to later-life sport requires attention to how bodily, socioeconomic, relational, and institutional conditions shape the opportunities available to older adults. Similar levels of expenditure, service access, or participation may coexist with different degrees of substantive freedom, particularly when resources cannot be adapted to individual circumstances or participation preferences.

### 3.2 Relevance to participatory sport consumption in later life

PSCILF concerns the sport-related resources through which older adults organise their own participation. Its later-life specificity lies in the interaction between these resources and heterogeneous bodily, socioeconomic, relational, and institutional circumstances. Functional changes, retirement transitions, previous sport experience, and changing social relationships may influence resource use in different ways across older adults. These conditions are neither universal to ageing nor exclusive to later life, but their interaction can shape the accessibility, adaptability, and continuity of sport-related participation.

The Capability Approach provides an appropriate analytical structure because it separates what resources are available from what an older adult can actually do with them. Facility membership, for example, provides formal access but does not establish whether the individual can reach the venue, manage the physical demands, understand instruction, adjust participation safely, or integrate attendance into everyday life. The substantive value of membership therefore depends on whether the service provides feasible opportunities that accommodate the individual’s bodily circumstances, participation preferences, and everyday commitments.

Conversion factors connect individual circumstances with the social and institutional organisation of sport consumption. Personal factors influence how older adults engage with available resources, while socioeconomic and relational conditions shape affordability, practical support, and continuity of participation. Environmental and service conditions determine whether resources can be accessed and adapted to individual needs. Sport services organised around uniform performance standards, purchasing power, or technological proficiency may restrict participation opportunities for consumers whose circumstances differ from those assumed by service providers. These restrictions arise through the interaction between individual characteristics and resource arrangements, allowing unequal capability to be examined without attributing limited participation solely to individual deficits.

Capabilities refer to the feasible opportunities generated through this conversion process, including opportunities to participate safely, acquire skills, regulate effort, exercise choice, maintain valued routines, and enter socially meaningful settings. Functionings arise when some of these opportunities are realised through sustained participation, everyday autonomy, social inclusion, psychological vitality, or other valued forms of living. For older consumers, capability expansion may therefore involve the opportunity to adapt participation as circumstances change while retaining access to activities and social relationships they value.

Agency remains central to this distinction. Capability expansion does not require continuous or maximised participation. An older adult can possess a meaningful opportunity and choose not to exercise it, while frequent participation may occur under prescription, pressure, or restricted alternatives. Participation frequency therefore cannot independently demonstrate capability expansion. Agency also requires meaningful influence over participation arrangements, including the ability to select suitable activities, adjust intensity, and decide whether and how participation continues. Service provision that offers formal access while allowing little practical choice may therefore support attendance without expanding substantive freedom.

Behavioural, motivational, and consumer perspectives provide insights into participation, persistence, resource acquisition, and service experience. The Capability Approach addresses the additional question of whether sport-related resources generate substantive opportunities under particular personal and institutional conditions. Its emphasis on conversion factors and agency enables PSCILF to be evaluated in relation to both individual participation experiences and the organisation of sport provision. This provides the theoretical basis for examining how accessible and adaptable services may expand older adults’ meaningful choices, while restrictive service arrangements may contribute to capability failure.

### 3.3 Capability-informed research in sporting contexts

Capability-informed sport research provides an established basis for this application. In Sport for Development and Peace, the approach has been used to challenge evaluations based only on programme provision or participation rates. Capability-informed scholarship has emphasised the need to evaluate the substantive opportunities available to participants and the social and institutional conditions shaping those opportunities (Svensson and Levine, 2017). Critical applications of the approach further highlight the importance of examining sporting provision within wider development and social conditions, rather than assuming that participation itself produces beneficial outcomes (Darnell and Dao, 2017).

Research has subsequently connected capability reasoning more closely with programme practice. Work drawing on a sport-for-development project in Vietnam showed how programme objectives, organisational arrangements, and participant agency can be examined together rather than assuming that participation itself produces developmental benefits (Dao and Smith, 2022). Related empirical analysis in the same national context demonstrated how capability concepts can be used to evaluate whether sporting provision creates meaningful opportunities associated with participation and social inclusion rather than simply documenting programme attendance (Dao and Darnell, 2022).

Capability analysis has also been applied beyond sport-for-development programmes. An 18-month ethnographic study in a multi-ethnic college in southwestern China found that the same sporting provision could be experienced as an externally imposed fitness obligation or as a valued part of everyday life, depending on participants’ circumstances, agency, and interpretation of the activity (Li et al., 2026). Although this study concerns college students, its findings illustrate how institutional expectations and individual agency may shape the capability value of sporting provision. Its relevance to PSCILF lies in the distinction between formally available participation and opportunities that individuals can exercise in accordance with their circumstances and values.

These applications establish the relevance of the Capability Approach to sporting opportunities, participant agency, and the institutional conditions of resource conversion. PSCILF extends this reasoning to the goods, services, technologies, and organised settings through which older adults access and sustain their own participation. Its capability value depends on whether these resources accommodate heterogeneous later-life circumstances and expand opportunities that older consumers can feasibly exercise and have reason to value.

## 4 Capability adaptation, evidence integration, and conceptual architecture of participatory sport consumption in later life

### 4.1 Capability adaptation to later-life sport consumption

The framework develops through theory adaptation, with the Capability Approach providing its primary analytical structure. Conceptual theory building can extend an established theoretical perspective by specifying its application to a new domain, clarifying relationships among constructs, and identifying the conditions under which those relationships hold (Jaakkola, 2020). In the present paper, the distinction among resources, conversion factors, capabilities, and functionings is applied to PSCILF, with particular attention to how later-life circumstances and sport-service arrangements shape substantive participation opportunities.

Three adaptations are required. First, sport-consumption resources extend beyond privately purchased goods. Facilities, services, technologies, professional instruction, community programmes, subsidised provision, and organised settings can all function as resources when they directly support older adults’ own participation. Their availability represents potential means for action rather than evidence of capability expansion. This broad understanding of resources enables commercial, public, and community-based provision to be examined within the same evaluative framework, while preserving differences in how consumers obtain and use these resources.

Second, resource convertibility occupies a central position. Similar services or technologies can generate different practical opportunities because their use is conditioned by health and functional capacity, socioeconomic resources, social relationships, environmental accessibility, technological competence, and service organisation. The adaptation therefore treats service design and institutional arrangements as conversion conditions alongside individual circumstances. Pricing structures, participation requirements, physical performance standards, and opportunities for service adaptation may expand or restrict the practical choices available to older consumers. Their influence depends on how these arrangements interact with heterogeneous later-life circumstances.

Third, observable participation remains analytically distinct from capability. Attendance, exercise frequency, or continued service use indicate realised behaviour, but they do not establish whether older adults possess meaningful alternatives, retain control over participation, or engage in activities they have reason to value. The framework consequently evaluates both the opportunities created by sport-related provision and older adults’ agency in deciding whether and how to exercise them. This preserves the distinction between resources, substantive freedom, and realised functionings, while allowing capability failure to be identified even when consumption or participation continues.

### 4.2 Integrating theoretical and empirical evidence

The Capability Approach provides the organising logic of the framework, while complementary literatures clarify psychological and relational dimensions of resource conversion. Self-determination research informs the interpretation of competence, autonomy, and volitional engagement within participation (Ryan and Deci, 2000). Social-participation scholarship supports the distinction between simple social contact and forms of participation involving interaction, recognition, and relational involvement (Levasseur et al., 2010). Active-ageing research provides the broader evaluative context in which autonomy, participation, inclusion, and later-life wellbeing acquire significance (Walker, 2002). These perspectives clarify how competence, connectedness, and valued later-life functionings may emerge within the resource-conversion process. The Capability Approach retains the primary evaluative role by distinguishing psychological and relational experiences from substantive opportunities and individual agency.

Empirical research informs the framework by identifying how sport-related resources operate within particular participation settings. Evidence from organised sport, exercise services, digital interventions, and group-based activities is used to examine the relationships among resource availability, participation conditions, and observed outcomes. Studies involving older populations provide contextual evidence concerning later-life resource use, while research involving other age groups is used where its conceptual or empirical findings inform the proposed relationships. This distinction avoids treating evidence from general sport participation as direct confirmation of capability building among older consumers.

Evidence concerning unequal access, heterogeneous participation experiences, and unsuccessful resource use is incorporated to identify potential restrictions in capability conversion. These restrictions may arise through the interaction between individual circumstances and service arrangements, including affordability, accessibility, participation requirements, and opportunities for adaptation. Such evidence informs the conceptualisation of capability failure without assuming that every instance of limited participation represents capability deprivation. The theoretical synthesis consequently distinguishes what existing studies establish empirically from the explanatory relationships proposed within the framework.

### 4.3 Consolidating the conceptual architecture

The conceptual architecture was developed by consolidating related constructs into a smaller number of theoretically connected dimensions. Sport-related goods, services, technologies, facilities, professional support, and organised settings were positioned as resources. Conditions affecting their effective use were organised into personal, socioeconomic and relational, and environmental and service conversion conditions. The latter domain includes organisational rules, participation standards, and service adaptability, allowing institutional restrictions to be examined as part of resource conversion rather than attributed solely to older adults’ individual characteristics.

A broader set of processes associated with bodily feedback, skill acquisition, progress recognition, choice, interaction, recognition, belonging, and identity was further consolidated into three forms of capability building. Mastery conversion captures the development of practical control through learning, feedback, skill acquisition, and recognised progress. Agency conversion concerns meaningful choice and practical control over the organisation of participation. Relational-identity conversion captures affiliation, recognition, belonging, and the development of socially viable roles through participation. Their contribution to capability expansion depends on whether the surrounding resource arrangements allow older adults to exercise these forms of practical, personal, and relational agency.

Competence and connectedness are positioned as proximal psychological and relational manifestations of these processes. Competence concerns the experience of acting effectively within available opportunities, while connectedness concerns relational embeddedness generated through continuing interaction and recognition. Neither construct is equated with capability itself, which refers to the substantive range of opportunities available to the individual. Capability expansion concerns the widening of feasible and valued opportunities, which may be realised through sustained participation, everyday autonomy, psychological vitality, social inclusion, and perceived quality of life. These functionings remain conditional on individual choice and circumstances. Restrictions arising from resource inequality or unsuitable service arrangements may limit substantive opportunities even when formal access or participation is maintained.

The resulting framework conceptualises PSCILF as a conditional process in which sport-related resources acquire capability value through their conversion into substantive opportunities. Personal circumstances and institutional arrangements jointly shape resource convertibility, while mastery, agency, and relational-identity processes explain how effective resource use may expand practical and socially valued opportunities. Competence and connectedness capture proximal manifestations of capability building, and later-life functionings represent possible realised outcomes. Capability failure occurs where restrictions in resource access, convertibility, or meaningful choice prevent the expansion of substantive opportunities.

## 5 Capability conversion in participatory sport consumption in later life

PSCILF can contribute to capability building when sport-related resources become feasible and valued opportunities for participation. Goods, services, technologies, facilities, professional support, and organised settings provide potential means for action, while their capability value depends on the conditions under which older adults can use them. The framework therefore focuses on resource convertibility rather than resource possession or participation frequency alone. In later life, resource convertibility may be shaped by changing bodily circumstances, socioeconomic resources, social relationships, and the adaptability of sport-related services. These conditions influence whether older consumers can maintain meaningful participation choices as their circumstances change.

Capability building is organised around three processes: mastery, agency, and relational-identity conversion. These processes may become psychologically and relationally visible through competence and connectedness and may contribute to valued later-life functionings. The relationships remain conditional because similar resources can generate different opportunities across individuals and settings, while restricted conversion may result in capability failure (Figure 1).

**Figure 1:**
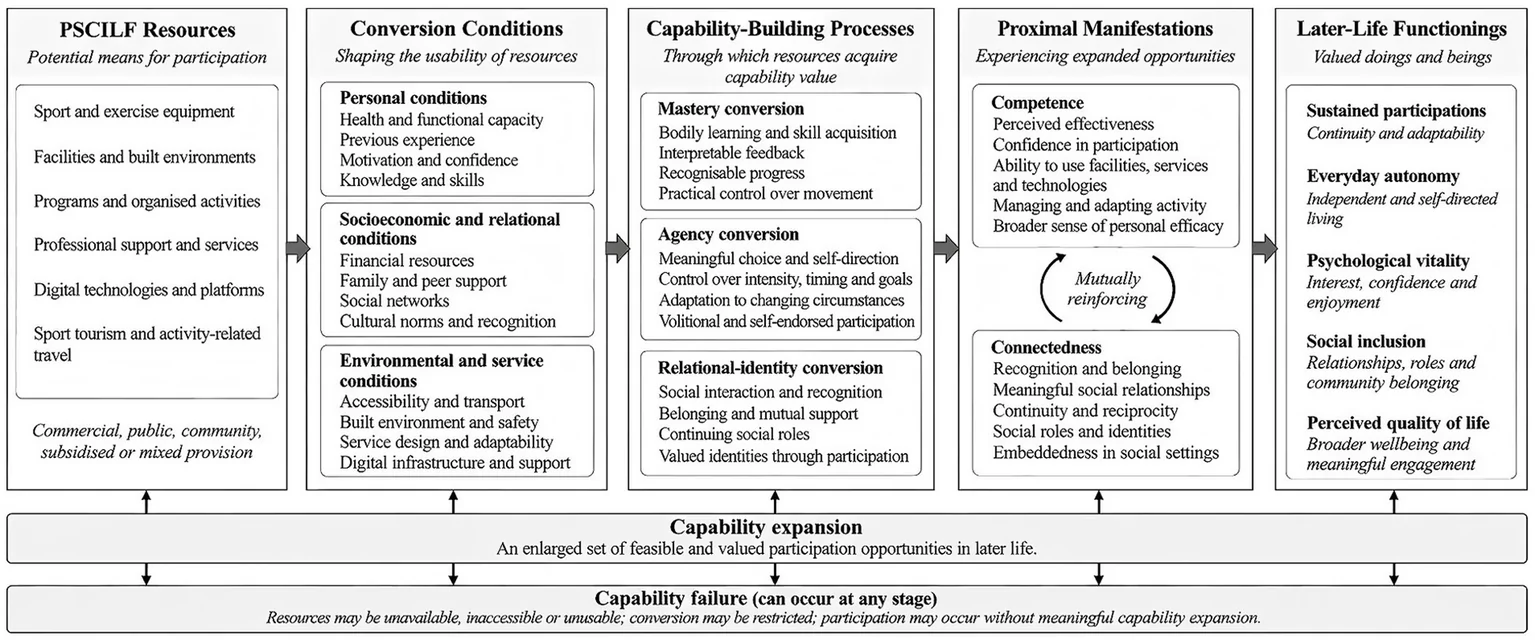
The capability-based framework of participatory sport consumption in later life. Sport-related resources constitute potential means for participation. Their conversion into valued opportunities depends on personal, socioeconomic and relational, and environmental and service conditions. Mastery, agency, and relational-identity conversion represent capability-building processes; competence and connectedness represent proximal psychological and relational manifestations. Capability failure may occur when resource access does not generate feasible and valued opportunities.

### 5.1 Resource convertibility

Sport-related resources include equipment, facility access, memberships, instruction, organised programmes, rehabilitation-oriented services, digital technologies, and other arrangements that directly support participation. Their provision may be commercial, public, community-based, or subsidised. Evidence from older adults in Wuhan, for example, shows variation in expenditure across sport products, participation-related services, and exercise-rehabilitation services according to socioeconomic and participation characteristics (Zhou et al., 2025). Such variation describes the resource environment but does not establish what individuals can accomplish through those resources.

Resource convertibility depends on how available provision interacts with older adults’ circumstances. A systematic review and thematic synthesis of qualitative studies examining older people’s perspectives on physical activity identified health conditions, fear of injury, previous experiences, professional support, and perceived activity suitability as factors influencing participation (Franco et al., 2015). These findings indicate that access to a sport-related resource may offer different practical opportunities according to an individual’s bodily condition, experience, and available support.

Digital provision provides a further example. An umbrella review and meta-meta-analysis of digital physical-activity interventions among older adults reported beneficial effects on physical activity, alongside variation in intervention characteristics and outcomes (Alley et al., 2024). Within PSCILF, these findings support examining whether digital resources can be used effectively under different individual and service conditions. The availability of a device or programme alone does not establish substantive participation opportunity.

Social and environmental conditions further influence resource conversion. A systematic review examining the association between social support and physical activity among older adults identified family and peer support as relevant to participation (Lindsay Smith et al., 2017). A systematic review and meta-analysis of built-environment correlates of older adults’ physical activity and walking identified features including recreational facilities, public open spaces, walkability, and transport (Barnett et al., 2017). These findings indicate that the practical value of sport-related resources depends partly on whether older adults can reach, use, and sustain participation within their everyday environments. Service affordability, accessibility, and adaptability further shape the substantive opportunities generated through resource use.

### 5.2 Capability-building processes

Three processes explain how effective resource use may expand practical opportunities. Mastery conversion concerns the development of practical control through bodily learning, skill acquisition, interpretable feedback, and recognised progress. An exercise and physical-activity position statement for older adults identifies appropriately designed aerobic, resistance, flexibility, and balance activities as relevant to maintaining or improving physical functioning (Chodzko-Zajko et al., 2009). Within PSCILF, such changes become capability relevant when older adults acquire skills and bodily understanding that enlarge their feasible participation options. A systematic review and meta-analysis of randomised controlled trials involving community-dwelling older adults found that wearable activity tracker-based interventions could improve physical activity, although effects across other physical outcomes were less consistent (Li et al., 2025). This evidence supports examining how interpretable feedback may facilitate mastery conversion, while the expansion of substantive opportunity remains a distinct theoretical proposition requiring empirical testing.

Agency conversion concerns meaningful choice and practical control over participation. A systematic review of exercise and physical-activity research grounded in self-determination theory found that autonomous motivation was associated with exercise participation and persistence (Teixeira et al., 2012). Although this evidence is not specific to older consumers, it supports the relevance of volitional engagement to sustained activity. The Capability Approach extends this explanation by examining whether meaningful participation alternatives are substantively available. For older adults, agency conversion may involve the ability to adjust activity type, intensity, timing, and support as bodily and everyday circumstances change. A service offering nominal consumer choice without feasible opportunities for adaptation may therefore support participation without expanding substantive freedom.

Relational-identity conversion concerns affiliation, recognition, belonging, and opportunities to occupy valued social roles. A mixed-methods systematic review of community-based group exercise interventions for older people identified social interaction, enjoyment, routine, and perceived benefits as relevant to programme adherence (Farrance et al., 2016). These findings suggest that organised participation can create conditions for recurring interaction and relational continuity. However, a scoping review of interventions addressing social connectedness and loneliness among older adults indicates that the relationship between social contact and meaningful connectedness requires attention to the nature of the intervention and its relational outcomes (O’Rourke et al., 2018). Within PSCILF, relational-identity conversion depends on whether repeated participation creates opportunities for recognition, belonging, and continuing social roles.

### 5.3 Competence and connectedness

Competence refers to perceived effectiveness in using available participation opportunities. A longitudinal study of older adults examined relationships among physical activity, self-efficacy, and self-esteem, providing evidence that participation and perceptions of personal effectiveness are related over time (McAuley et al., 2005). Within PSCILF, competence may be expressed through movement confidence, skill execution, exercise regulation, or effective use of sport-related services. These experiences may support continued engagement, although perceived competence does not establish whether affordable, accessible, and meaningful participation opportunities are available.

Connectedness refers to relational embeddedness developed through continuing interaction, recognition, reciprocity, and belonging. The convoy model of social relations explains how interpersonal networks provide support, identity, and continuity across the life course (Antonucci et al., 2014). Although the model does not specifically examine sport consumption, it provides a theoretical basis for understanding how recurring participation settings may become part of older adults’ relational networks. Within PSCILF, connectedness develops when interactions with peers, instructors, or programme communities acquire continuing relational significance.

Competence and connectedness may reinforce one another when practical confidence facilitates participation in shared settings and relational support encourages continued learning. However, a longitudinal study of middle-aged and older American adults examined bidirectional relationships between physical activity and loneliness, indicating that their association should be understood in relation to both temporal and social conditions (Surkalim et al., 2024). Within PSCILF, the development of competence does not necessarily produce connectedness, while social contact alone does not establish relational capability. Their relationship depends on the opportunities for meaningful participation and interaction provided by particular resource configurations.

### 5.4 Later-life functionings and capability failure

Capability expansion may contribute to sustained participation, everyday autonomy, psychological vitality, social inclusion, and perceived quality of life. These are potential functionings rather than predetermined consequences of sport consumption. Sustained participation may involve adaptation of activity type, intensity, setting, or support as bodily and social circumstances change. Everyday autonomy concerns practical control over participation and movement, while social inclusion concerns continued access to relationships, shared settings, and recognised roles. Older adults’ accounts of ageing well consistently emphasise independence, social relationships, psychological outlook, health, and meaningful activity (Bowling, 2008).

Capability failure remains possible even where resources and participation are present. Digital technologies may be formally available but difficult to use; commercial services may provide extensive options but remain economically inaccessible; organised programmes may maintain attendance while allowing little meaningful choice or social recognition. Research on digital exclusion among older adults demonstrates that technological access can coexist with limited skills, support, and social inclusion (Seifert et al., 2021). Although this research does not directly evaluate sport consumption, it provides a relevant context for examining digital conversion barriers within PSCILF. Similar restrictions may arise when commercial services remain unaffordable, programmes assume uniform bodily capabilities, or participation arrangements provide limited opportunities for adaptation and meaningful choice. Capability failure therefore concerns restrictions in substantive opportunity, including those arising from the interaction between individual circumstances and service organisation.

PSCILF is consequently conceptualised as a potential resource for capability expansion whose value depends on the conversion of sport-related provision into feasible and valued opportunities. Mastery, agency, and relational-identity processes explain how effective resource use may expand practical and socially valued participation options, while competence and connectedness represent proximal manifestations of capability building. Later-life functionings reflect possible realisations of expanded capability, whereas capability failure identifies conditions under which resource availability or participation remains disconnected from substantive freedom.

## 6 Resource conversion, capability failure, and empirical testing of the framework

### 6.1 Resource conversion as the theoretical contribution

The principal contribution of the framework lies in explaining how resource conversion shapes the relationship between PSCILF and valued later-life functionings. Sport-consumption research can identify what individuals purchase, access, and use, while physical-activity research can establish whether participation occurs and how frequently it is sustained. These forms of evidence provide limited insight into whether available resources expand the range of opportunities that older adults can feasibly exercise and personally value.

Capability-informed sport research has demonstrated the importance of distinguishing programme provision from the opportunities participants are actually able to exercise. Sporting environments generate different developmental consequences according to participant agency, institutional arrangements, and the social conditions surrounding participation (Darnell and Dao, 2017). The Capability Approach similarly directs evaluation toward what people are substantively able to do and become rather than toward provision or participation alone (Svensson and Levine, 2017). Applying this distinction to PSCILF makes it possible to examine how sport-related resources interact with heterogeneous later-life circumstances and institutional arrangements to shape substantive participation opportunities.

The framework also separates substantive opportunity from its psychological and relational manifestations. Competence indicates perceived effectiveness within participation, while connectedness reflects relational embeddedness and recognition. Both may accompany capability building, but neither defines capability itself. This distinction allows resource availability, substantive opportunity, psychosocial experience, and realised functioning to be examined as related but conceptually distinct dimensions.

The framework further locates capability differences within the interaction between individual circumstances and the organisation of sport provision. Pricing, accessibility, participation requirements, and service adaptability influence whether similar resources generate comparable opportunities for different older consumers. Mastery, agency, and relational-identity conversion explain how effective resource use may expand practical control, meaningful choice, and socially valued participation. The Capability Approach thus provides an evaluative basis for examining both individual experiences and institutional conditions that facilitate or restrict capability expansion in later life.

### 6.2 Capability failure as a boundary condition

The framework is conditional because resource availability does not guarantee capability expansion. Older adults differ in access to resources and in their ability to convert formally similar resources into valued opportunities. These differences may arise from personal circumstances, unequal resource distribution, and service arrangements that do not accommodate heterogeneous participation needs. Capability failure therefore requires attention to the relationship between individual conversion conditions and the institutional organisation of sport consumption.

Digital participation illustrates this distinction. Access to devices or online services can coexist with limited skills, confidence, support, or meaningful use. Research on digital exclusion among older adults demonstrates that technological availability may remain embedded in wider forms of social and digital inequality (Seifert et al., 2021). Although this research did not specifically examine sport consumption, it provides a relevant basis for considering how digitally mediated sport services may remain inaccessible despite formal availability. Digital exclusion illustrates how resource access can coexist with restricted substantive opportunity when technological requirements exceed the support available to particular consumers.

Institutional arrangements may further restrict resource conversion. Market-oriented sport provision can make access and continued participation dependent on purchasing power, while standardised participation requirements may privilege particular forms of physical performance and independence. Where services are organised around assumptions of able-bodied performance, continuous improvement, or uniform exercise capacity, older consumers with different bodily circumstances or participation preferences may encounter restricted opportunities. These arrangements can be examined through the concepts of neoliberalism and ableism, particularly where individual responsibility and prescribed bodily standards shape access, service design, and expectations of participation.

The Capability Approach locates these restrictions within social and institutional conversion conditions. A consumer may have access to a facility while lacking suitable activity options, affordable support, or meaningful influence over participation arrangements. Resource availability can therefore coexist with limited substantive freedom when service design does not accommodate individual circumstances. Capability failure may arise through the interaction between consumers’ resources and the institutional conditions governing their use.

Capability failure can also occur within continued participation. Attendance does not establish meaningful choice, recognition, or practical control. Critical sport scholarship has cautioned against assuming that participation generates beneficial outcomes simply because sport is involved (Coakley, 2015). An older adult may continue using a service while experiencing poor fit, restricted choice, weak social recognition, or demands that cannot be sustained.

Capability failure is therefore defined as a condition in which available sport-related resources do not enlarge the feasible and valued opportunities available to an older adult. Its occurrence may reflect unequal resource distribution, restrictive conversion conditions, or participation arrangements that limit meaningful choice. This interpretation extends the framework’s explanatory scope to institutional conditions that may reproduce inequalities in later-life sport consumption.

### 6.3 Integrated theoretical propositions

Four propositions capture the principal relationships within the framework.

*Proposition 1*: Resource-conversion proposition. Sport-related consumption resources contribute to capability expansion when personal, socioeconomic and relational, and environmental and service conditions allow them to be converted into feasible and valued participation opportunities.
*Proposition 2*: Capability-building proposition. Effective resource conversion can expand practical and socially valued opportunities through mastery, agency, and relational-identity processes.
*Proposition 3*: Proximal-manifestation proposition. Competence and connectedness represent proximal psychological and relational manifestations of capability building and are associated with the realisation of valued later-life functionings.
*Proposition 4*: Capability-failure proposition. Persistent restrictions in resource conversion can prevent sport-related resources from expanding substantive opportunities and can contribute to inequalities in later-life participation.

The four propositions specify the relationships through which PSCILF resources may acquire capability value. Resource convertibility shapes the opportunities available to older consumers, while capability-building processes explain how effective resource use may support competence, connectedness, and valued later-life functionings. Individual and institutional restrictions may interrupt these relationships and contribute to capability failure (Table 1).

**Table 1:** Theoretical propositions of the capability-based framework of participatory sport consumption in later life.

Proposition	Theoretical relationship	Empirical focus
P1 Resource conversion	Sport-related resources support capability expansion when conversion conditions make their use feasible and valuable.	Access, affordability, usability, accessibility, social support
P2 Capability building	Mastery, agency, and relational-identity processes explain how effective resource use can expand practical opportunities.	Learning, feedback, meaningful choice, recognition, belonging
P3 Proximal manifestations	Competence and connectedness reflect psychological and relational manifestations of capability building associated with later-life functionings.	Perceived effectiveness, connectedness, autonomy, sustained participation, inclusion
P4 Capability failure	Restrictive conversion conditions can prevent available resources from expanding substantive opportunity.	Constrained choice, unused resources, discontinuation, exclusion, unequal conversion

### 6.4 Empirical testing and age-inclusive sport consumption implications

Empirical testing should preserve the distinctions among resources, conversion conditions, capability-building processes, competence and connectedness, and later-life functionings. Measuring PSCILF only through expenditure, service use, or participation frequency would obscure the central theoretical question. Studies should assess what resources are available, whether they can be effectively used, and whether older adults possess feasible and valued alternatives for participation.

Longitudinal research can examine how resource conversion and capability-building processes develop through repeated participation and whether competence and connectedness are associated with subsequent changes in valued functionings. Comparative studies across commercial, public, community-based, and digital settings can further examine how pricing, accessibility, service adaptability, and institutional requirements influence capability expansion among older consumers with different bodily and socioeconomic circumstances.

Cases of capability failure are particularly important for examining institutional restrictions. Research involving unused memberships, discontinued services, inaccessible programmes, or participation under constrained choice can help identify whether limited capability expansion arises from resource inequality, individual conversion conditions, or the organisation of sport provision.

Quantitative approaches can examine conditional and indirect relationships among the proposed constructs once appropriate measurement models have been established, with structural equation modelling providing one possible strategy for testing theoretically specified relationships (MacCallum and Austin, 2000). Qualitative and mixed-methods research remains important because feasibility, meaningful choice, recognition, and valued opportunity partly depend on how older adults interpret their own circumstances.

## 7 Conclusion

PSCILF focuses on the resources through which older adults organise and sustain sport and exercise participation. The Capability Approach explains how resource convertibility shapes the substantive opportunities available to older consumers under heterogeneous personal, social, and institutional conditions. Mastery, agency, and relational-identity processes may support competence, connectedness, and valued later-life functionings, while restrictive conversion conditions can result in capability failure. The framework provides a basis for evaluating age-inclusive sport consumption policies and services according to their capacity to expand meaningful choice and feasible participation opportunities across diverse later-life circumstances.

## References

[ref1] Alley S. Waters K. Parker F. Peiris D. L. I. H. K. Fien S. Rebar A. . (2024). The effectiveness of digital physical activity interventions in older adults: a systematic umbrella review and meta-meta-analysis. Int. J. Behav. Nutr. Phys. Act. 21:144. doi: 10.1186/s12966-024-01694-4, 39696583 PMC11658456

[ref2] Antonucci T. C. Ajrouch K. J. Birditt K. S. (2014). The convoy model: explaining social relations from a multidisciplinary perspective. The Gerontologist 54, 82–92. doi: 10.1093/geront/gnt118, 24142914 PMC3894851

[ref3] Barnett D. W. Barnett A. Nathan A. Van Cauwenberg J. Cerin E. . (2017). Built environmental correlates of older adults’ total physical activity and walking: a systematic review and meta-analysis. Int. J. Behav. Nutr. Phys. Act. 14:103. doi: 10.1186/s12966-017-0558-z PMC5547528 28784183

[ref4] Bennett E. V. Hurd Clarke L. Wolf S. A. Dunlop W. L. Harden S. M. Liu Y. . (2018). Older adults' experiences of group-based physical activity: a qualitative study from the ‘GOAL’ randomized controlled trial. Psychol. Sport Exerc. 39, 184–192. doi: 10.1016/j.psychsport.2018.08.017

[ref5] Bowling A. (2008). Enhancing later life: how older people perceive active ageing? Aging Ment. Health 12, 293–301. doi: 10.1080/13607860802120979, 18728941

[ref6] Brady S. D’Ambrosio L. A. Felts A. Rula E. Y. Kell K. P. Coughlin J. F. (2020). Reducing isolation and loneliness through membership in a fitness program for older adults: implications for health. J. Appl. Gerontol. 39, 301–310. doi: 10.1177/0733464818807820, 30392420 PMC7005930

[ref7] Caspersen C. J. Powell K. E. Christenson G. M. (1985). Physical activity, exercise, and physical fitness: definitions and distinctions for health-related research. Public Health Rep. 100, 126–131, 3920711 PMC1424733

[ref8] Chen G. Janke M. C. Liechty T. Wong J. D. West S. T. Son J. S. . (2023). Sport participation for adults aged 50 + years: a socioecological analysis. Int. J. Aging Hum. Dev. 97, 354–373. doi: 10.1177/00914150221143958, 36464642

[ref9] Chodzko-Zajko W. Proctor D. Fiatarone Singh M. Minson C. Nigg C. Salem G. . (2009). Exercise and physical activity for older adults. Med. Sci. Sports Exerc. 41, 1510–1530. doi: 10.1249/MSS.0b013e3181a0c95c, 19516148

[ref10] Coakley J. (2015). Assessing the sociology of sport: on cultural sensibilities and the great sport myth. Int. Rev. Sociol. Sport 50, 402–406. doi: 10.1177/1012690214538864

[ref11] Dao M. S. Darnell S. C. (2022). Exploring Vietnamese sport for development through the capabilities approach: a descriptive analysis. Sport Soc. 25, 1987–2008. doi: 10.1080/17430437.2021.1900828

[ref12] Dao M. Smith T. (2022). The capability approach as a conceptual bridge for theory-practice in sport-for-development. J. Glob. Sport Manag. 7, 310–326. doi: 10.1080/24704067.2019.1703117

[ref13] Darnell S. Dao M. (2017). Considering sport for development and peace through the capabilities approach. Third World Themat.: TWQ J. 2, 23–36. doi: 10.1080/23802014.2017.1314772

[ref14] Doyle J. P. Filo K. Lock D. Funk D. C. McDonald H. (2016). Exploring PERMA in spectator sport: applying positive psychology to examine the individual-level benefits of sport consumption. Sport Manag. Rev. 19, 506–519. doi: 10.1016/j.smr.2016.04.007

[ref15] Eime R. M. Young J. A. Harvey J. T. Charity M. J. Payne W. R. (2013). A systematic review of the psychological and social benefits of participation in sport for adults: informing development of a conceptual model of health through sport. Int. J. Behav. Nutr. Phys. Act. 10:135. doi: 10.1186/1479-5868-10-135, 24313992 PMC4028858

[ref16] Farrance C. Tsofliou F. Clark C. (2016). Adherence to community based group exercise interventions for older people: a mixed-methods systematic review. Prev. Med. 87, 155–166. doi: 10.1016/j.ypmed.2016.02.037, 26921655

[ref17] Franco M. R. Tong A. Howard K. Sherrington C. Ferreira P. H. Pinto R. Z. . (2015). Older people's perspectives on participation in physical activity: a systematic review and thematic synthesis of qualitative literature. Br. J. Sports Med. 49, 1268–1276. doi: 10.1136/bjsports-2014-094015, 25586911

[ref18] Funk D. C. Mahony D. F. Havitz M. E. (2003). Sport consumer behavior: assessment and direction. Sport Mark. Q. 12, 200–205. doi: 10.1177/106169340301200402

[ref19] Gayman A. M. Fraser-Thomas J. Dionigi R. A. Horton S. Baker J. (2017). Is sport good for older adults? A systematic review of psychosocial outcomes of older adults’ sport participation. Int. Rev. Sport Exerc. Psychol. 10, 164–185. doi: 10.1080/1750984X.2016.1199046

[ref20] Geidne S. Ericson H. Quennerstedt M. (2026). What older adults find meaningful in organised sport participation: proposing the HeRO model. Front. Sports Act. Living 8:1760024. doi: 10.3389/fspor.2026.1760024 PMC13333586 42440514

[ref21] Howat G. Assaker G. (2016). Outcome quality in participant sport and recreation service quality models: empirical results from public aquatic centres in Australia. Sport Manag. Rev. 19, 520–535. doi: 10.1016/j.smr.2016.04.002

[ref22] Hyun M. Jordan J. S. (2020). Athletic goal achievement: a critical antecedent of event satisfaction, re-participation intention, and future exercise intention in participant sport events. Sport Manag. Rev. 23, 256–270. doi: 10.1016/j.smr.2019.01.007

[ref23] Jaakkola E. (2020). Designing conceptual articles: four approaches. AMS Rev. 10, 18–26. doi: 10.1007/s13162-020-00161-0

[ref24] Jenkin C. R. Eime R. M. Westerbeek H. O’Sullivan G. van Uffelen J. G. Z. (2017). Sport and ageing: a systematic review of the determinants and trends of participation in sport for older adults. BMC Public Health 17:976. doi: 10.1186/s12889-017-4970-8, 29273036 PMC5741887

[ref25] Jenkin C. R. Eime R. M. Westerbeek H. van Uffelen J. G. Z. (2018). Sport for adults aged 50+ years: participation benefits and barriers. J. Aging Phys. Act. 26, 363–371. doi: 10.1123/japa.2017-0092, 28952860

[ref26] Kim A. C. H. Park S. H. Kim S. Fontes-Comber A. (2020). Psychological and social outcomes of sport participation for older adults: a systematic review. Ageing Soc. 40, 1529–1549. doi: 10.1017/S0144686X19000175

[ref27] Ko Y. J. Pastore D. L. (2004). Current issues and conceptualizations of service quality in the recreation sport industry. Sport Mark. Q. 13, 158–166. doi: 10.1177/106169340401300306

[ref28] Levasseur M. Richard L. Gauvin L. Raymond É. (2010). Inventory and analysis of definitions of social participation found in the aging literature: proposed taxonomy of social activities. Soc. Sci. Med. 71, 2141–2149. doi: 10.1016/j.socscimed.2010.09.041, 21044812 PMC3597625

[ref29] Li R. Li Y. Wang L. Li L. Fu C. Hu D. . (2025). Wearable activity tracker–based interventions for physical activity, body composition, and physical function among community-dwelling older adults: systematic review and meta-analysis of randomized controlled trials. J. Med. Internet Res. 27:e59507. doi: 10.2196/59507, 40179387 PMC12006780

[ref30] Li Y. Ramli M. S. Zhang Y. Kawashima K. (2026). A fitness obligation or indispensable pastime? A capability-informed inquiry into the developmental impacts of tiyu(体育) on college students in multi-ethnic China. Sport Educ. Soc. 31, 615–630. doi: 10.1080/13573322.2024.2426630

[ref31] Lindsay Smith G. Banting L. Eime R. O’Sullivan G. van Uffelen J. G. Z. (2017). The association between social support and physical activity in older adults: a systematic review. Int. J. Behav. Nutr. Phys. Act. 14:56. doi: 10.1186/s12966-017-0509-8, 28449673 PMC5408452

[ref32] MacCallum R. C. Austin J. T. (2000). Applications of structural equation modeling in psychological research. Annu. Rev. Psychol. 51, 201–226. doi: 10.1146/annurev.psych.51.1.201, 10751970

[ref33] Malkowski O. S. Harvey J. Townsend N. P. Kelson M. J. Foster C. E. M. Western M. J. . (2025). Correlates and determinants of physical activity among older adults of lower versus higher socio-economic status: a systematic review and meta-analysis. Int. J. Behav. Nutr. Phys. Act. 22:83. doi: 10.1186/s12966-025-01775-y PMC12183859 40545526

[ref34] Masayuki Y. Makoto N. (2016). Innovative sport consumption experience: an empirical test in spectator and participant sports. J. Appl. Sport Manag. 8:6024. doi: 10.18666/JASM-2016-V8-I1-6024

[ref35] McAuley E. Elavsky S. Motl R. W. Konopack J. F. Hu L. Marquez D. X. (2005). Physical activity, self-efficacy, and self-esteem: longitudinal relationships in older adults. J. Gerontol. Series B 60, P268–P275. doi: 10.1093/geronb/60.5.P268, 16131621

[ref36] McDonald M. A. Milne G. R. Hong J. (2002). Motivational factors for evaluating sport spectator and participant markets. Sport Mark. Q. 11, 100–113. doi: 10.1177/106169340201100206

[ref37] Nussbaum M. C. (2011). Creating Capabilities the Human Development Approach. Cambridge: Harvard University Press.

[ref38] O’Rourke H. M. Collins L. Sidani S. (2018). Interventions to address social connectedness and loneliness for older adults: a scoping review. BMC Geriatr. 18:214. doi: 10.1186/s12877-018-0897-x, 30219034 PMC6139173

[ref39] Oettle L. Greiner J. (2025). *Coping with poverty and social exclusion: promoting capabilities through long-term sports participation*. Social Exclusion 13:8341. doi: 10.17645/si.8341

[ref40] Richard V. Piumatti G. Pullen N. Lorthe E. Guessous I. Cantoreggi N. . (2023). Socioeconomic inequalities in sport participation: pattern per sport and time trends – a repeated cross-sectional study. BMC Public Health 23:785. doi: 10.1186/s12889-023-15650-7, 37118691 PMC10141913

[ref41] Robeyns I. (2005). The capability approach: a theoretical survey. J. Hum. Dev. 6, 93–117. doi: 10.1080/146498805200034266

[ref42] Ryan R. M. Deci E. L. (2000). Self-determination theory and the facilitation of intrinsic motivation, social development, and well-being. Am. Psychol. 55, 68–78. doi: 10.1037//0003-066x.55.1.68, 11392867

[ref43] Sánchez-Santos J. M. Rungo P. Lera-López F. (2024). Building social capital through sport engagement: evidence for adults aged 50 years and older. Ageing Soc. 44, 403–428. doi: 10.1017/S0144686X22000046

[ref44] Sauter A. Curbach J. Rueter J. Lindacher V. Loss J. (2019). German senior citizens’ capabilities for physical activity: a qualitative study. Health Promot. Int. 34, 1117–1129. doi: 10.1093/heapro/day077, 30272159

[ref45] Seifert A. Cotten S. R. Xie B. (2021). A double burden of exclusion? Digital and social exclusion of older adults in times of COVID-19. J. Gerontol. Series B 76, e99–e103. doi: 10.1093/geronb/gbaa098, 32672332 PMC7454901

[ref46] Sen A. (1999). Development as Freedom. Oxford: Oxford University Press.

[ref47] Sivaramakrishnan H. Gucciardi D. F. McDonald M. D. Quested E. Thogersen-Ntoumani C. Cheval B. . (2024). Psychosocial outcomes of sport participation for middle-aged and older adults: a systematic review and meta-analysis. Int. Rev. Sport Exerc. Psychol. 17, 398–419. doi: 10.1080/1750984X.2021.2004611

[ref48] Solis-Navarro L. Gismero A. Fernández-Jané C. Torres-Castro R. Solá-Madurell M. Bergé C. . (2022). Effectiveness of home-based exercise delivered by digital health in older adults: a systematic review and meta-analysis. Age Ageing 51:afac243. doi: 10.1093/ageing/afac243, 36346736 PMC9642810

[ref49] Stenner B. J. Buckley J. D. Mosewich A. D. (2020). Reasons why older adults play sport: a systematic review. J. Sport Health Sci. 9, 530–541. doi: 10.1016/j.jshs.2019.11.003, 33308804 PMC7749227

[ref50] Surkalim D. L. Clare P. J. Eres R. Gebel K. Bauman A. E. Ding D. (2024). Exercise to socialize? Bidirectional relationships between physical activity and loneliness in middle-aged and older American adults. Am. J. Epidemiol. 193, 996–1001. doi: 10.1093/aje/kwae001, 38319704 PMC11228862

[ref51] Suzuki N. (2017). A capability approach to understanding sport for social inclusion: agency, structure and organisations. Social Inclusion 5, 150–158. doi: 10.17645/si.v5i2.905

[ref52] Svensson P. G. Levine J. (2017). Rethinking sport for development and peace: the capability approach. Sport Soc. 20, 905–923. doi: 10.1080/17430437.2016.1269083

[ref53] Teixeira P. J. Carraça E. V. Markland D. Silva M. N. Ryan R. M. (2012). Exercise, physical activity, and self-determination theory: a systematic review. Int. J. Behav. Nutr. Phys. Act. 9:78. doi: 10.1186/1479-5868-9-78, 22726453 PMC3441783

[ref54] Walker A. (2002). A strategy for active ageing. Int. Soc. Secur. Rev. 55, 121–139. doi: 10.1111/1468-246X.00118

[ref55] Yamakita M. Kanamori S. Kondo N. Kondo K. (2015). Correlates of regular participation in sports groups among Japanese older adults: JAGES cross–sectional study. PLoS One 10:e0141638. doi: 10.1371/journal.pone.0141638, 26512895 PMC4626107

[ref56] Zhou C. Ji Q. Shi X. Liu H. Chen Y. (2025). Associations between physical activity participation and different types of sports consumption expenditures among older adults: a cross-sectional study. Front. Public Health 13:1713320. doi: 10.3389/fpubh.2025.1713320 PMC12753444 41480068

[ref57] Zimmer C. McDonough M. H. Hewson J. Toohey A. Din C. Crocker P. R. E. . (2021). Experiences with social participation in group physical activity programs for older adults. J. Sport Exerc. Psychol. 43, 335–344. doi: 10.1123/jsep.2020-0335, 34167084

